# Supplementary material for: A novel Axin2 knock‐in mouse model for visualization and lineage tracing of WNT/CTNNB1 responsive cells
Source: Genesis. 2020 Jul 9;58(9):e23387. doi: 10.1002/dvg.23387 (PMC7539917; doi:10.1002/dvg.23387)
Supplement: Supplementary file 12 — TABLE S1 Mendelian inheritance of the 3′ Axin2 P2A‐rtTA3‐T2A‐3xNLS‐SGFP2 allele. TABLE S2: Non‐mendelian inheritance in Axin2 CreERT2 knock‐in mice. TABLE S3: Primer sequences. [file DVG-58-e23387-s012.docx]

**Supplementary Table 1. Mendelian inheritance of the 3’ *Axin2^P2A-rtTA3-T2A-3xNLS-SGFP2^* allele**

Homozygous *Axin2^P2A-rtTA3-T2A-3xNLS-SGFP2^* mice are born at the expected mendelian ratios from heterozygous intercrosses on a C57BL/6 background.
(Chi-square test, P=0.61 for both sexes combined).

|  | *Axin2^P2A-rtTA3-T2A-3xNLS-sGFP2^*  **(Heterozygous intercross on a C57Bl/6 background)** | |
| --- | --- | --- |
|  | Expected | Observed |
|  |  |  |
| WT (wildtype) | 8.25 | 6 |
| HET (heterozygous) | 16.5 | 17 |
| HOM (homozygous) | 8.25 | 10 |
| Total | 33 | 33 |

**Supplementary Table 2. Non-mendelian inheritance in *Axin2^CreERT2^* knock-in mice**

By design, all 5’ *Axin2* knock-in alleles generated to date (*Axin2^lacZ^*, *Axin2^mTq2^*, *Axin2^CreERT2^*) are predicted to disrupt endogenous *Axin2* expression (Supplementary Figure 1). However, mice heterozygous for *Axin2* were previously reported to be phenotypically normal (Lustig et al., 2001; Van Amerongen, Bowman, & Nusse, 2012; Zeng & Nusse, 2010).

Although we have bred *Axin2^CreERT2^* mice (targeting performed in ES cells from a 129/Sv background) without any trouble at multiple different animal facilities in back-crosses to both C57BL/6J and FVB/N, we noticed that even heterozygous animals were present upon weaning at sub-mendelian ratios after rederivation onto a C57BL/6JRj background (A, Chi-square test, P=0.00077 for both sexes combined). This coincided with the presence of a skull phenotype in some heterozygous animals, resembling that described for homozygous *Axin2^lacZ^* mice by (Yu et al., 2005). Sub-mendelian inheritance of the knock-in allele remained apparent during the first three generations of heterozygous backcrossing of the mice from A onto FVB/N (B, Chi-square test, P=3.5x10^-6^ for both sexes combined), after which wildtype and heterozygous mice were again present upon weaning at the expected ratios (C, Chi-square test, P=0.85 for both sexes combined).

These data were collected between September 2014 and April 2018 as part of our regular colony maintenance and genotyping efforts. Note that this information has been used to update the strain description on the Jackson laboratory website in August 2017. We would also like to point out that Jackson labs now reports that homozygous *Axin2^CreERT2^* mice are born “at a significantly reduced frequency (~5% in heterozygous crosses)”, as stated on their website (<https://www.jax.org/strain/018867>, accessed 5 June 2020). These observations suggest that the 5’ *Axin2^CreERT2^* knock-in allele is haploinsufficient on a C57BL/6JRj background. At present we have no explanation for this observation, nor do we have an explanation for why neither of these effects (haploinsufficiency and reduced numbers of homozygous animals) have been observed in *Axin2^lacZ^* mice, which carry a knock-in cassette at the exact same location.

|  | **A** | |  | **B** | |  | **C** | |
| --- | --- | --- | --- | --- | --- | --- | --- | --- |
|  | *Axin2^CreERT2^*  **(C57BL/6J and C57BL/6JRj backcross)** | |  | *Axin2^CreERT2^*  **(FVB backcross, generation F1-F3)** | |  | *Axin2^CreERT2^*  **(FVB backcross, generation F4-F6)** | |
|  | Expected | Observed |  | Expected | Observed |  | Expected | Observed |
| WT;WT (wildtype) | 42.5 | 58 |  | 41 | 62 |  | 14.5 | 14 |
| WT;KI (heterozygous) | 42.5 | 27 |  | 41 | 20 |  | 14.5 | 15 |
| Total | 85 | 85 |  | 82 | 82 |  | 29 | 29 |

**Supplementary Table 3. Primer sequences**

PCR genotyping:

| **Strain** | **Primer** | **Description** | **Sequence (5’ to 3’)** | **details** | **Product** |
| --- | --- | --- | --- | --- | --- |
| *Axin2^P2A-rtTA3-T2A-3xNLS-SGFP2^* | RVA 1786  RVA 1787  RVA 1789 | Ozgene 01F (WT PCR)  Ozgene 02R (common)  Ozgene 04F (mut PCR) | CGAGCTTTCCTGTCTTCTCTTAAG  GAATATTTCGTGGCTGTTGCGTAG  CAACAGCCACAACGTCTATATCAC | T_ann_ = 52°C,  30 cycli | Wild type allele = 257 bp  Knock-in allele = 461 bp |
| *tetO-Wnt5a* | RVA 6  RVA 7 | Fw  Rv | ACAAAGACGATGACGACAAGC  CGCACCTTCTCCAATGTACTG | T_ann_ = 55°C,  30 cycli | Transgenic product = 200 bp |
| *tetO-Cre(Bjd)* | RVA 2422  RVA 2423 | Fw  Rv | TTACAGATGCACATATCGAGG  TAACCCAGTAGATCCAGAGG | T_ann_ = 58°C,  30 cycli | Transgenic product = 490 bp |
| *Rosa26^mTmG^* | RVA 284  RVA 285  RVA 286 | WT Fw  WT Rv  Mut Rv | CTCTGCTGCCTCCTGGCTTCT  CGAGGCGGATCACAAGCAATA  TCAATGGGCGGGGGTCGTT | T_ann_ = 58°C,  35 cycli | Wild type allele = 330 bp  Knock-in allele = 250 bp |

qRT-PCR:

| **Gene** | **Primer** | **Description** | **Sequence (5’ to 3’)** |
| --- | --- | --- | --- |
| *Axin2* | RVA 12  RVA 13 | Axin2 Fw  Axin2 Rv | AGCTGGTTGTCACCTACT  CAGGCAAATTCGTCACTCG |
| *Rpl13a* | RVA 413  RVA 414 | Rpl13a Fw  Rpl13a Rv | CCCTCCACCCTATGACAAGA  GCCCCAGGTAAGCAAACTT |
| *SGFP2* | RVA 1784  RVA 1785 | GFP Fw  GFP Rv | CCAGGAGCGCACCATCTTCT  TGCCGTTCTTCTGCTTGTCG |
| *rtTA3* | RVA 276  RVA 277 | rtTA Fw  rtTA Rv | AGGCGAGTCATGGCAAGA  GGGAGAAGCCTTGCTGACA |
